# From Conventional Methods to Innovation: Caffeine and Chlorogenic Acid Extraction and Quantification in the Rise of Smart and Green Techniques—A Systematic Review

**DOI:** 10.3390/molecules31111890

**Published:** 2026-06-01

**Authors:** Shady H. Awwad, Lara M. Nasereddin, Ola Al-Tamimi, Ahmad Q. Daraosheh, Ali Elrashidi, Lydia Abu Al-Shayeb, Mais Shannag, Beisan A. Mohammad, Reem Issa, Mahmoud S. Abu-Samak

**Affiliations:** 1 Department of Pharmaceutical Chemistry and Pharmacognosy, Faculty of Pharmacy, Applied Science Private University (ASU), Amman 11937, Jordan; laranasereddin@gmail.com; 2 Vitamin D+ Allostatic Load: DAL+ Research Group, Faculty of Pharmacy, Applied Science Private University (ASU), Amman 11937, Jordan; bmohammad@fcms.edu.sa (B.A.M.); m_abusamak@asu.edu.jo (M.S.A.-S.); 3 Department of Pharmaceutical Sciences, School of Pharmacy, University of Jordan, Amman 11942, Jordan; olatameemi@gmail.com; 4 Department of Chemistry, College of Arts and Sciences, University of Petra, Amman 11196, Jordan; adaraosheh@uop.edu.jo; 5 Electrical Engineering Department, College of Engineering, University of Business and Technology, Jeddah 23435, Saudi Arabia; a.elrashidi@ubt.edu.sa; 6 Engineering Mathematics Department, Faculty of Engineering, Alexandria University, Alexandria 21544, Egypt; 7 Department of Pharmaceutical Sciences and Pharmaceutics, Faculty of Pharmacy, Applied Science Private University (ASU), Amman 11937, Jordan; l_alshayeb@asu.edu.jo; 8 Department of Clinical Pharmacy and Therapeutics, Faculty of Pharmacy, Applied Science Private University (ASU), Amman 11937, Jordan; maisalshannaq@gmail.com; 9 Pharmaceutical Sciences Department-PharmD Program, Fakeeh College for Medical Sciences, Jeddah 21461, Saudi Arabia, Fakeeh Care Group; 10 Department of Basic Pharmaceutical Sciences, Faculty of Pharmacy, Middle East University, Amman 11831, Jordan; r.issa@meu.edu.jo

**Keywords:** caffeine, chlorogenic acid, extraction, green chemistry, quantification

## Abstract

Caffeine and chlorogenic acid are among the most extensively investigated bioactive compounds in coffee, tea, and other plant-derived products due to their noteworthy physical, nutritional, and industrial relevance. Caffeine is primarily acknowledged for its central nervous system stimulant activity, whereas chlorogenic acid, a phenolic ester, contributes antioxidant, anti-inflammatory, and metabolic health benefits. This review was conducted according to the PRISMA guidelines in order to systematically compile and summarize the extraction and analytical conditions reported for caffeine and CGAs in different matrices and to provide a structured comparison among the reported studies. All studies focusing on the extraction and/or quantification of caffeine and chlorogenic acids in several matrices were considered eligible. Three independent electronic searches were performed using PubMed, Science.gov, and BASE to identify relevant articles. Extraction of data was conducted independently by four authors based on consistent selection and extraction criteria. One hundred and twenty-five studies were identified. The results were summarized in tables including several parameters. Conventional extraction techniques, including aqueous and organic solvent-based methods, have formed the foundation for separating caffeine and chlorogenic acids. However, rising interest in green and sustainable technologies has shifted attention towards advanced approaches such as ultrasound-assisted extraction and microwave-assisted extraction. These methods not only enhance extraction yields and reduce processing times but also align with environmental and safety concerns in the modern food and pharmaceutical industries. For quantification, high-performance liquid chromatography equipped with ultraviolet or mass spectrometric detection remains the benchmark, offering precision and reproducibility in different matrices. This review sheds light on recent advances and ongoing research in the extraction and quantification of caffeine and chlorogenic acid in different types of matrices. Continued innovation in green extraction technologies and robust quantification methods is essential for supporting scientific research applications.

## 1. Introduction

Caffeine and chlorogenic acids (CGAs) are considered two of the most prominent bioactive compounds that are commonly found in coffee, tea, plants, and foods. These compounds have gained remarkable attention due to their numerous chemical and physical properties, as well as their physiological effects [1,2]. The global relevance of caffeine and CGAs is underscored by their widespread consumption, primarily through coffee, which constitutes the main dietary source of both compounds.

Caffeine, known as 1,3,7-trimethylxanthine, is a natural purine alkaloid belonging to the methylxanthine family, typically appearing in its pure form as an odorless, white, bitter-tasting solid in fine needle or powder form [3]. It functions as a central nervous system stimulant that reduces drowsiness and enhances alertness, exerting effects on the central nervous, cardiovascular, gastrointestinal, respiratory, and renal systems. Physiological responses include increased heart rate, elevated plasma glucose and free fatty acid levels, cerebral vasoconstriction, and diuretic effects through enhanced renal blood flow and glomerular filtration rate. However, high doses (~1 g) may induce nervousness, insomnia, and tremors. Caffeine has also been linked to a probable reduction in Parkinson’s disease and type 2 diabetes, though it may elevate blood pressure and increase the risk of pregnancy loss [4]. Its psychostimulant activity is primarily mediated through antagonism of adenosine receptors, thereby modulating alertness and cognitive function [5].

CGAs, a group of esters formed between trans-hydroxycinnamic acids and quinic acid, mainly include 5-caffeoylquinic acid (5-CQA), 3-caffeoylquinic acid (3-CQA), 4-caffeoylquinic acid (4-CQA), and feruloylquinic acids (FQAs). These compounds exhibit noteworthy antioxidant and anti-inflammatory activities, contributing to protection against chronic diseases such as cardiovascular diseases, type 2 diabetes, and certain neurodegenerative disorders. Additionally, CGAs have been implicated in weight reduction and demonstrated antimicrobial properties against various bacteria and fungi, acting as prebiotics by enhancing beneficial gut microbiota and inhibiting intestinal non-heme iron absorption [6]. Table 1 presents a comparative analysis of the major characteristics of caffeine and CGAs [7,8,9,10].

Beyond their distinct bioactivities, both caffeine and CGAs substantially influence coffee’s physical qualities such as aroma, flavor, and mouthfeel owing to their high concentrations and chemical complexity within the coffee bean [11,12]. It has been demonstrated that the method of extraction itself is an essential factor in determining the final concentration of key bioactive compounds such as caffeine and CGAs. The rise of green extraction techniques is closely tied to growing environmental awareness, stricter regulations, and advances in green chemistry. Furthermore, green extraction aims to reduce the impact of using traditional extraction methods while maintaining or even improving efficiency. A previous study highlighted that by systematically adjusting various process parameters, it is possible to significantly influence the recovery of these molecules [13]. The findings showed that aspects of the physical preparation of the material and the design of the extraction system can each contribute to considerable variations in the final concentrations of these compounds. This work establishes that a deliberate and controlled approach to extraction is essential for modulating the presence of these important bioactive substances [13].

Certain chemical compounds can serve as effective markers for discriminating between plant varieties. Martin et al. (1998) utilized a chemometric approach to differentiate between two green varieties based on their chemical composition [14]. The research specifically identified caffeine and CGAs, along with other compounds, as key descriptors for this classification. The findings, derived from principal component and cluster analysis, indicated that these specific components were particularly powerful in distinguishing between the two varieties [14]. This work highlighted the utility of analyzing the concentrations of compounds like caffeine and CGAs as a reliable method for botanical classification and quality control.

Caffeine is recognized for its pesticidal properties and its role in mediating interactions between plants and insects. CGAs are a class of phenolic compounds that are also involved in these plant–insect interactions [15]. These phytochemicals are abundant in young leaves of certain plants, which are often the most vulnerable to insect attacks. A decline in CGA levels in plant leaves can occur due to infestation by certain insects, and this reduction may favor further infestation by other generalist insects. Therefore, their concentrations are key factors in a plant’s natural defense mechanisms against various insect pests [15].

For the accurate quantification and characterization of these compounds in both green and roasted coffee, advanced analytical techniques are indispensable. High-performance liquid chromatography (HPLC) and nuclear magnetic resonance (NMR) spectroscopy are extensively employed, providing critical insights into the chemical transformations occurring during processing and enabling precise determination of compound concentrations [16,17,18,19]. Furthermore, effective extraction methodologies are crucial prerequisites for accurate quantification, influencing the yield and purity of the isolated bioactive compounds for subsequent analysis and potential application. A comprehensive understanding of extraction methodologies, quantification techniques, and the impact of different roasting intensities on the thermal stability and degradation pathways of key bioactive compounds such as caffeine and CGAs is paramount for optimizing coffee processing. Such knowledge is not only crucial for achieving desired sensory characteristics but also for maximizing the retention or beneficial transformation of compounds associated with health benefits.

Caffeine and CGAs are among the most broadly investigated bioactive components in coffee, tea, and plant-based foods. The extraction methods have evolved from conventional techniques toward green and smart technologies, but the published literature is fragmented, with no clear synthesis comparing efficiency and analytical performance. Their results are heterogeneous and difficult to compare. Despite extensive research, no consensus exists on optimal extraction and quantification methodologies, due to wide methodological variability, matrix effects, and inconsistent validation practices. Moreover, existing reviews are often narrative, focusing on either extraction or quantification with limited attention to green and smart techniques. A systematic review is needed to critically synthesize existing analytical approaches, identify methodological gaps, provide a structured comparison, and guide future research and standardization efforts. Therefore, this review aims to synthesize and critically evaluate the current scientific literature concerning the extraction and quantification of caffeine and CGAs.

## 2. Results and Discussion

**Figure 1:**
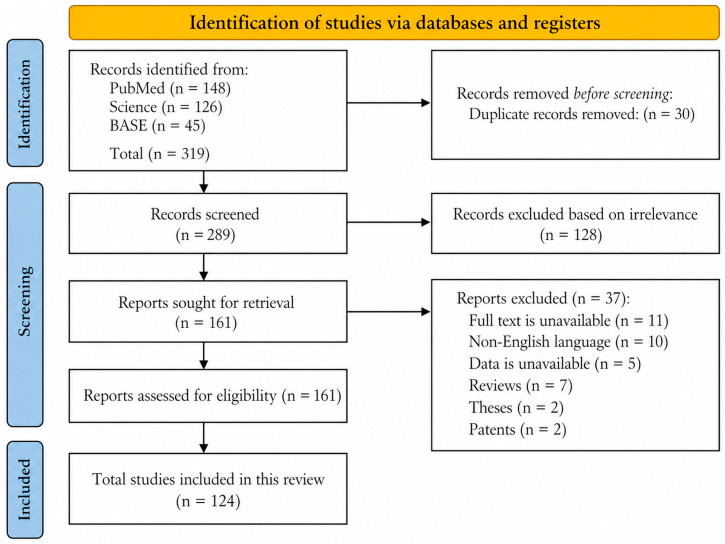
PRISMA flow diagram showing the number of articles identified and selected in this systematic review.

This search yielded a total of 319 articles, of which 124 articles were included, and 195 were excluded based on several criteria. The process of selecting the articles included in this systematic review was guided by specific inclusion and exclusion criteria, as demonstrated in Figure 1. The studies included in this systematic review reveal substantial methodological heterogeneity, largely attributable to the use of numerous analytical platforms such as HPLC, liquid chromatography mass spectrometry (LC-MS), gas chromatography mass spectrometry (GC-MS), and NMR, as well as a wide range of extraction techniques. Among these platforms, HPLC equipped with different types of detectors emerged as a commonly employed analytical technique for qualitative and quantitative analysis, likely due to its wide availability, cost-effectiveness, operational simplicity, and strong suitability for routine analysis of known compounds. Nevertheless, some studies employed LC-MS, as it offers superior sensitivity and selectivity, enabling the detection of trace-level compounds and facilitating structural elucidation. In other studies, GC-MS proved to be effective for the analysis of volatile and thermally stable constituents. These instrumental variations among the reported studies inherently influence metabolite detection, identification, and quantification, thereby contributing to inconsistencies across study findings. In parallel, the selected studies exhibited diversity in extraction methods, including variations in solvent polarity, extraction time, solvent volume, and temperature. Most studies utilized liquid–liquid extraction (LLE) due to its versatility, simplicity, and cost-effectiveness. Recent research has also demonstrated a shift towards green extraction methods such as ultrasound-assisted extraction (UAE), microwave-assisted extraction (MAE), and deep eutectic solvent (DES) extraction. As a result, discrepancies observed between these studies are likely driven by methodological differences rather than biological or compositional variation. Despite these limitations, the combined use of diverse analytical techniques and extraction approaches can be viewed as complementary, enhancing overall chemical coverage and providing a more comprehensive understanding of the matrices studied. However, diversity also limits direct comparability and underscores the need for greater methodological standardization in future research.

### 2.1. Extraction of Caffeine and Chlorogenic Acid

Caffeine and CGAs are commonly extracted from *Coffea arabica*, along with numerous other species and subspecies of the *Coffea* genus. Several of these, including *C. canephora*, *C. liberica*, *C. humilis*, *C. mannii*, *C. charrieriana*, *C. kapakata*, and *C. anythonyi*, have been documented to contain promising caffeine and CGA concentrations [20,21]. These compounds can be extracted from coffee beans (green or roasted) [5,22,23], leaves [20], flowers [24,25], cherries [26], and endosperms [27,28]. Additionally, they can be found in coffee silverskin [29,30], husk (pulp) [31,32], refuse [33,34,35], espresso spent coffee grounds [13,27], coffee tree dry branches [36,37], and coffee bagasse [36].

Nevertheless, the genus *Coffea* is one of several plant groups known to contain notable amounts of caffeine and CGA. Many of these include green tea leaves [38,39], aerial parts of Greek mountain tea (*Sideritis raeseri*) [17], leaves and aerial parts of yerba mate (*Ilex paraguariensis*) [40,41,42], Ceylon tea leaves [38], and cocoa (husk, pod husk, and shells) [31]. However, some plants contain only one of the two key compounds; for example, Gardenia leaves (*Gardenia jasminoides*) contain CGAs only [43,44].

Despite the presence of several caffeine and CGA sources, the amount of compounds extracted highly depends on the extraction method and the applied parameters such as the plant part used, coffee origin, harvest time, roasting and grinding degree, solvent type and volume, brewing ratio, extraction time, pressure, and temperature [45,46,47]. Prior to extraction, coffee beans may be stored in liquid nitrogen or a deep freeze to facilitate grinding [46,48]. Several extraction techniques have been reported, including solid–liquid extraction (SLE), green extraction, and LLE. LLE is a conventional and widely used method due to its simplicity and ability to efficiently extract caffeine, which exhibits moderate polarity and good solubility in organic solvents. However, LLE lacks selectivity and often leads to the co-extraction of other compounds, making it less suitable for accurate CGA isolation, which is more polar and water-soluble [49,50]. On the other hand, SLE provides enhanced selectivity and cleaner extracts by utilizing tailored sorbents that can differentially retain caffeine and CGAs, thereby improving purification and analytical precision, particularly in chromatographic systems [51]. Meanwhile, green extraction techniques have gained increasing attention due to their reduced solvent consumption, shorter extraction times, and improved extraction efficiency. These methods are particularly advantageous for CGAs, as they enhance the extraction of thermolabile and polar compounds while maintaining good yields of caffeine [52]. Overall, while LLE remains suitable for routine caffeine extraction, SLE offers superior selectivity for simultaneous analysis, and green extraction techniques provide a more efficient and sustainable approach for extracting both caffeine and CGAs from coffee matrices.

Ethanol and water are recognized as the most effective solvents for enhancing the extraction of bioactive compounds [37,53]. Several other solvents have also been applied, including dichloromethane, hexane, and methanol [54,55]. Additionally, food-grade gases such as N_2_, CO_2_, N_2_O, and Ar have shown significant effects on the physicochemical properties of coffee, mainly depending on the type of gas [56]. However, ethanol and water remain the most commonly employed solvents due to their non-toxic nature and approval for use in food manufacturing. Furthermore, they are often combined as a mixture to improve the extraction efficiency of polar compounds [24]. There has been debate concerning optimal extraction conditions. For dry coffee bagasse, using 30% ethanol at 30 °C for 30 min of sonication yields the highest phenolic content and antioxidant capacity [37]. This suggests that a lower ethanol concentration and mild temperature are sufficient to extract the remaining bioactive compounds from a processed matrix. Meanwhile, for green coffee beans, regardless of temperature, a solvent mixture of ethanol and water (40:60) achieves the best extraction results [57]. This indicates that, for green coffee beans, the optimal condition is a higher proportion of water, indicating that a more polar solvent system is more effective for extracting abundant polar compounds such as CGAs from a less processed matrix. Thus, the optimal conditions vary depending on the nature of the raw material and the chemical profile of target compounds. While both cases aim to maximize extraction efficiency, the differences in solvent polarity and extraction parameters reflect the need to tailor conditions to the specific physicochemical characteristics of the sample, rather than applying a universal extraction protocol. Despite the many established methods, the literature agrees that there is no “optimal” extraction method, but rather each technique excels within its own parameters [58].

#### 2.1.1. Liquid–Solid Extraction (LSE)

Several LSE techniques have been documented, including solid-phase extraction (SPE) [54], vortex-assisted extraction (VAE) [36], Soxhlet extraction [59], and reflux extraction [48]. Additional methods include dynamic maceration [36], anti-nucleation extraction [34], and traditional coffee brewing techniques, such as AeroPress [58], Caffè Firenze [56], espresso [12], French Press [58], Moka pot [12], and the V60 brewing method [58].

A typical LSE process occurs by dissolving ground plant material in a solvent (e.g., acetone, chloroform, ethanol, ethyl acetate, methanol, or water) or in their mixtures at a wide range of temperatures: room temperature (RT), warm, or boiling [24,53,60]. It involves three main steps: (I) Ground coffee absorbs water during brewing. (II) Coffee compounds migrate from the grounds to the water. (III) The extract is separated from the spent solids [58]. This method is used to extract phenolic compounds, regardless of the plant part used [32]. A critical factor that determines the efficiency of LSE is the degree of grinding, particularly when using green coffee. Therefore, fine-ground coffee is crucial for improving extraction efficiency [48].

#### 2.1.2. Solid-Phase Extraction (SPE)

SPE is a type of LSE that involves using a solid stationary phase, also called a solid-phase extractor, such as an ODS C18 cartridge. The process involves moving the liquid extract after preparation through the extractor, which selectively retains the target compounds through binding interactions. This purifies the extract, making it suitable for further chromatographic analysis [15,54]. According to a previous study, the concentrations of caffeine, CGAs, caffeic acid, and related methylxanthines were determined in coffee leaf extracts [15]. Coffee leaves were collected, dried, and finely ground prior to extraction. The powdered samples were extracted with methanol and incubated in a water bath for phytochemical extraction. Subsequently, the extracts were further purified using a C-18 SPE cartridge. The resulting extracts were diluted with water and analyzed by HPLC. Alkaloids (e.g., caffeine and methylxanthines) were detected at 272 nm, while phenolic compounds (CGAs and caffeic acid) were monitored at 320 nm. Caffeine concentrations ranged from 3.08 to 1486.04 ppm, whereas CGA levels ranged from 240.55 to 922.95 ppm [15].

#### 2.1.3. Vortex-Assisted Extraction (VAE)

VAE is a type of LSE that utilizes a vortex mixer to disperse the extraction solvent into the sample, allowing for a faster extraction process by enhancing mass transfer. VAE may involve the use of two immiscible liquids. Hence, it can also be considered an LLE technique [36]. Around twenty compounds were characterized in several coffee by-product samples [36]. The coffee samples were oven-dried under air circulation and subsequently ground using an analytical mill. Three extraction approaches were evaluated using ethanol–water (70:30%) as the extraction solvent. One of these was vortex-assisted extraction, which was performed with a magnetic stirrer at 3000 rpm for 30 min, using approximately 500 mg of sample and 5 mL of the extraction solvent in a 25 mL Falcon tube. In addition, biphasic liquid–liquid extraction systems were assessed by introducing 5 mL of n-heptane to the extraction mixture. This approach enabled the simultaneous generation of hydroethanolic and n-heptane fractions, which were subsequently analyzed by UHPLC-PDA/UV and GC-MS, respectively. The extraction yields ranged from 4.47% to 36.00%.

#### 2.1.4. Soxhlet Extraction

Soxhlet extraction involves washing the ground material with a heated solvent. ground green coffee beans were extracted in four cycles using heated methanol at 14 arbitrary units in a Büchi B-811 extraction system (Büchi, Switzerland), followed by a reflux washing step [48]. However, when the extract was examined using high-performance size exclusion chromatography (HPSEC), results showed insufficient peak separation of CGAs, which was explained by the degradation of CGAs during Soxhlet extraction [48]. Soxhlet extraction could be used to defat coffee. Additionally, Soxtherm Soxhlet extraction system and an organic solvent were used, e.g., dichloromethane, to defat coffee prior to further extraction [54].

#### 2.1.5. Reflux Extraction

Reflux extraction is a conventional technique that uses a heated, volatile solvent to extract the desired compounds. It operates by continuously heating the solvent to its boiling point. Its main advantages include reproducibility and suitability for exhaustive extraction, particularly for relatively stable compounds such as caffeine. Furthermore, it does not require specialized equipment and is widely accessible, making it a standard method in many laboratories. However, it has several drawbacks: it is time-consuming, requires large volumes of organic solvents, and involves prolonged exposure to high temperatures, which may lead to the degradation of thermolabile compounds such as CGAs. Additionally, its lack of selectivity can result in the co-extraction of unwanted components, necessitating further purification steps. When this method was compared to nonconventional methods, the latter showed superior performance, yielding four times higher caffeine concentrations and CGA concentrations 7.5 times higher than those obtained using the conventional reflux-based approach [59]. These methods improve extraction efficiency and yield through enhanced mass transfer. These techniques are particularly advantageous for extracting CGAs, as they can be optimized to operate under mild or controlled conditions, thereby minimizing thermal degradation.

#### 2.1.6. Dynamic Maceration

Dynamic maceration is an SLE method that involves submerging plant material in a suitable solvent. This is often followed by magnetic stirring to enhance extraction efficiency [36]. Approximately twenty compounds were characterized across various coffee by-products. The coffee samples were oven-dried, finely ground, and subsequently subjected to extraction as described in Section 2.1.3. Three extraction approaches were evaluated using ethanol–water (70:30%) as the extraction solvent. One of these was dynamic maceration, which was performed with a magnetic stirrer at 1400 rpm and 30 °C for 30 min, using approximately 500 mg of sample and 5 mL of the extraction solvent in a 3.0 cm internal diameter beaker. Finally, the hydroethanolic and n-heptane fractions were analyzed by UHPLC-PDA/UV and GC-MS, respectively.

#### 2.1.7. Anti-Nucleation Extraction

Anti-nucleation is an approach that involves the use of supercooling-facilitating (SCF) materials to inhibit or delay the ice nucleation process (freezing). This approach reduces the ability of water molecules to organize into ice crystals by removing or inactivating ice-nucleating agents [61]. This is typically achieved by using SCFs that promote supercooling. Briefly, SCFs lower the supercooling point of water. The extraction process is done by heating plant material with water at 80 °C for 1 h, followed by ultrafiltration with a specific molecular weight cutoff. Fractions with molecular weights below the cutoff are pH-adjusted, subjected to solvent fractionation with ethyl acetate, and dried. In the case of coffee refuse, the resulting extract showed high levels of caffeine and CGAs (173 and 62.3 μg/mL, respectively) due to ethyl acetate fractionation. Extracts with SCF properties are advertised to protect vegetables from frost damage [34].

#### 2.1.8. Green and Smart Extraction

Green extraction methods minimize environmental impact while improving the safety and efficiency of chemical processes. They reduce or eliminate the use of toxic organic solvents, lower energy consumption, and generate less waste, making them more sustainable than conventional extraction techniques. These methods also help protect human health and preserve the integrity of sensitive bioactive compounds, leading to higher-quality extracts. Several green extraction methods have been documented, including UAE [62], MAE [63], supercritical fluid extraction (SFE) [40,64], pressurized hot water extraction (PHWE) [24], and DES extraction [31,59]. Among these, UAE has been found to provide the highest yields of caffeine and CGAs [57].

##### Ultrasound-Assisted Extraction (UAE)

UAE is a green technique that utilizes an ultrasonication bath to enhance the recovery of bioactive compounds while reducing extraction time, solvent consumption, and energy use [12,65]. During the extraction, ultrasound energy creates physical effects such as erosion, capillarity, fragmentation, detexturation, and sonoporation [65]. Several studies have employed UAE to extract caffeine and CGAs from various plant types and parts. The extraction efficiency of UAE with dynamic maceration was compared on coffee refuse (husk, pulp, and silverskin). The results demonstrated the superiority of UAE [36]. Furthermore, this extraction method was used to extract bioactive compounds from coffee leaves [20], coffee beans [65], and *I. paraguariensis* leaves [62]. UAE can also be combined with other extraction techniques to reduce extraction time. For example, UAE was combined with cold brewing to enhance efficiency by shortening the extraction duration. However, this reduction resulted in lower caffeine and CGA content compared to static cold brews [65].

##### Microwave-Assisted Extraction (MAE)

MAE is an important green extraction technique that uses microwave energy to rapidly heat the solvent and plant material above the solvent’s boiling point, thereby facilitating the efficient release of bioactive compounds. Its importance lies in its ability to significantly reduce extraction time, solvent consumption, and energy usage while increasing extraction efficiency and yield. The feasibility of using MAE was evaluated on roasted coffee beans to generate a high-yield extract; their results demonstrated that MAE significantly improved extraction yields compared to conventional household methods [63].

##### Supercritical Fluid Extraction (SFE)

SFE is a green extraction technique because it provides high efficiency, selectivity, and minimal environmental impact by using non-toxic solvents, most commonly carbon dioxide. Its significance lies in its ability to produce high-purity extracts without leaving hazardous solvent residues, leading to its widespread adoption in the food, pharmaceutical, and natural product industries. In this technique, carbon dioxide is brought to its supercritical state by applying elevated pressure and temperature, where it exhibits both gas-like and liquid-like properties. Supercritical CO_2_ penetrates the plant material easily and dissolves target compounds, which are then separated by reducing the pressure, allowing the CO_2_ to return to gaseous form and leaving behind the extracted compounds. Antioxidants were extracted from spent coffee grounds and husks by applying CO_2_ and a co-solvent. However, when the results were compared to conventional extraction methods (reflux and ultrasound), conventional methods outperformed SFE in terms of total antioxidant yield [64].

##### Pressurized Hot Water Extraction (PHWE)

PHWE, also referred to as subcritical water extraction, is a green extraction technique that uses water instead of organic solvents, making it safe, inexpensive, and environmentally friendly. Its importance lies in reducing toxic solvent use while effectively extracting a wide range of bioactive compounds such as phenolics and flavonoids. PHWE was identified as a high-efficiency extraction approach; specifically, it was determined to be the optimal method for extracting bioactive compounds from coffee flowers [24].

##### Deep Eutectic Solvents (DESs)

DESs are valuable in green extraction as they offer a sustainable, low-toxicity alternative to conventional organic solvents; they are typically biodegradable, cost-effective, and derived from natural components. Their significance lies in their tunability, as their chemical composition can be tailored to selectively extract specific bioactive compounds—such as flavonoids, phenolics, and alkaloids—from plant matrices. For instance, This method was utilized to extract caffeine from coffee pulp, cocoa, and pod husks. Generally, the results demonstrated both the sustainability and high extraction capacity of DESs for biomolecules, while also facilitating the formation of molecular complexes that are unattainable through conventional approaches [31]. The major characteristics of green extraction techniques are summarized in Table 2 [66,67,68,69].

#### 2.1.9. Liquid–Liquid Extraction

CGAs were extracted from commercial ground roasted coffee using an LLE approach [70]. The procedure involved brewing the coffee with deionized water, followed by filtration and cooling. Subsequently, the brewed coffee was processed with dichloromethane using a liquid–liquid extractor and then dried. The results identified nine CGAs, including three caffeoylquinic acids, three feruloylquinic acids, and three dicaffeoylquinic acids. Among these, 5-CQA was found in the highest concentration [70].

### 2.2. Quantification of Caffeine and Chlorogenic Acid

Separation, identification, structural analysis, and characterization are all qualitative aspects that have been extensively studied in natural extracts in general and specifically for caffeine and CGAs. Hence, several methods have been developed to achieve optimal caffeine and CGA profiles. Analyses have been conducted on a variety of matrices, including coffee leaf extracts [20], coffee and willow bark extracts, coffee by-product extracts [34,53], coffee bean extracts (green or roasted) [16], coffee flower extracts [24], green tea leaf extracts [39], and *Ilex paraguariensis* leaf extracts [41], as well as sunflower hull extracts (*Helianthus annus*) [71], *Camellia sinensis* leaf extracts [72], and extracts from *Hydrocotyle sibthorpioides*, *Centella asiatica*, and *Amaranthus viridis* [73].

Various analytical methods are used to analyze green coffee. For example, HPLC, liquid chromatography with tandem mass spectrometry (LC-MS/MS), and Fourier transform mid-infrared attenuated total reflectance spectroscopy (FT–MIR–ATR) are commonly employed to quantify compounds in coffee samples. Meanwhile, polyphenol levels can be determined using HPLC-diode-array detector (DAD), ultra-performance liquid chromatography-mass spectrometry (UPLC–MS), micro-Raman spectroscopy, or Fourier-transform–ion cyclotron resonance–mass spectrometry (FT–ICR–MS) [57].

A comparative study was conducted to evaluate high-performance thin-layer chromatography (HPTLC), high-performance capillary electrophoresis (HPCE), high-speed acousto-optical tunable filter near-infrared spectrometry (AOTF-NIR), and HPLC as potential methods for quantifying CGA content in biological samples [74]. Overall, HPTLC was discouraged due to its low accuracy and limited sensitivity, as it requires confirmation with GC and UHPLC prior to use. In contrast, HPCE and AOTF-NIR are prohibitively expensive for routine laboratory-scale research. Consequently, HPLC was identified as the most recommended method due to its repeatability, selectivity, and sensitivity [74]. However, UV/Vis spectroscopy may outperform HPLC in terms of simplicity and accessibility [74].

#### 2.2.1. Liquid Chromatography–Electrochemical Detection (LC-EC)

CQAs were determined in water extracts of coffee leaves [20]. Separation was performed using a developed LC-EC approach in isocratic elution mode. Total run time was 25 min, with a flow rate of 0.8 mL/min and an injection volume of 20 µL. These conditions were found to be optimal for a six-month study without any loss of sensitivity. An electrochemical (EC) detector was employed due to its previously demonstrated high sensitivity for detecting readily oxidized CQAs. This method was validated using liquid chromatography–high-resolution mass spectrometry (LC-HRMS) [20].

To ensure peak purity and identity, and to enable accurate retention time comparison, target analytes and standards were also analyzed using LC-quadrupole time-of-flight mass spectrometry (LC-QTOF-MS) in gradient elution mode, allowing precise determination of molecular masses. Total run time was 28 min, with a flow rate of 0.50 mL/min, and the analysis was performed at 55 °C. The LC–EC results were further confirmed using a separate calibration curve for CQA isomers obtained by LC–QTOF–MS. Overall, peak overlaps observed in LC–EC were confirmed by LC–QTOF–MS. However, it was also reported that LC–EC could detect only readily oxidized compounds, which represents a limitation of the developed method [20].

#### 2.2.2. Liquid Chromatography–Mass Spectrometry (LC-MS)

Another study on the identification and characterization of caffeine and CGAs in extracts from coffee leaves, grape stems, and gardenia leaves [44]. The two compounds were characterized using UHPLC–MS to develop chemical fingerprints of the three extracts. Overall, both compounds were detected in the coffee leaf extract, whereas neither was detected in the grape stem extract. In contrast, only CGAs were detected in the gardenia leaf extract [44].

A specific method for the characterization of caffeine and CGAs was developed [75]. Identification of the phenolic profile was performed using Liquid Chromatography–Electrospray Ionization–Ultra-High Resolution–Quadrupole Time-of-Flight–Mass Spectrometry (LC-ESI-UHR-QTOF-MS) in gradient mode, with MS parameters set to positive ionization. This step was crucial for improving the accuracy of compound identification [75].

Meanwhile, the quantitative profile was determined using HPLC coupled with a photodiode array (PDA) detector. Separation was conducted on a reversed-phase column using gradient elution at a flow rate of 1 mL/min and a temperature of 25 °C, with an injection volume of 20 µL. Overall, the authors reported that the selection of the coffee blend significantly influences both the qualitative and quantitative profiles of bioactive compounds [75].

#### 2.2.3. High-Performance Liquid Chromatography with UV-Visible Detection (HPLC-UV/Vis)

Numerous studies have documented the use of HPLC for the quantification of caffeine and CGAs, with most methodological variations arising from the coupling of HPLC with different detectors. For instance, Ludwig et al. (2014) utilized a PDA to determine caffeine and CQA content in roasted, ground coffee beans [76]. A similar HPLC-PDA configuration was employed by Cruz et al. (2012) to quantify CGA isomers [27], as well as by Kawahara et al. (2017) to evaluate the anti-nucleation activity of coffee extracts [34]. Furthermore, HPLC-DAD was employed to quantify the concentrations of caffeine and CGAs in spent coffee grounds [35].

Phenolics and lactones were identified in regular and decaffeinated coffee using HPLC-UV. To detect CGAs, the ESI source was operated in negative ion and Single Ion Monitoring modes, with identity confirmation achieved via LC-MS. The results revealed a significant depletion in CGA levels in decaffeinated samples, a finding attributed to the decaffeination process [77].

Other researchers have integrated multiple chromatographic platforms to characterize these compounds. For instance, HPLC-UV was employed for CGA analysis, while LC-MS/MS was utilized to analyze caffeine and quantify both bioactives within a randomized pharmacokinetic trial. Their findings demonstrated that both chromatographic methods provided high efficiency, linearity, and sensitivity [5].

Additionally, nonvolatile compounds were semi-quantified, including caffeine and CGA, using HPLC-UV in gradient elution mode. For volatile profiles, however, the researchers employed headspace solid-phase microextraction coupled with GC-MS (HS-SPME-GC-MS). The study concluded that both the extraction method and the temperature are pivotal factors in determining the volatile profiles of the coffee samples [45].

The analytical profile of CQA and caffeine in *Ilex paraguariensis* was investigated [78]. HPLC-PDA was utilized for the separation and determination of phenolic acid levels using a binary convex gradient mode. Quantification was performed to ensure that the concentration ranges within the samples were fully encompassed by the calibration, while an HPLC-ESI/MS system was employed for data acquisition [78].

In a different approach, an accelerated HPLC-DAD procedure coupled with multivariate curve resolution–alternating least squares (MCR-ALS) was developed [16]. This decomposition method is designed to resolve overlapping signals in a sample, thereby allowing for accurate quantification of CGA and caffeine in green coffee beans. The proposed approach represents a valid alternative to standard methods, as it relies on the posterior resolution of profiles into individual compounds; consequently, it does not require fully resolved peaks at the baseline [16].

A method using HPLC equipped with a variable wavelength detector (HPLC-VWD) was developed to analyze caffeine and CGAs. Analysis was performed at 270 nm for caffeine and 345 nm for CGAs; the method was further validated for linearity, repeatability, within-laboratory reproducibility, limits of detection, and quantification. Additionally, specificity was confirmed through the stability of retention time [13].

#### 2.2.4. High-Performance Liquid Chromatography-Corona Detector (HPLC-CAD)

An HPLC-CAD method was developed to compare the bioactive compounds present in green coffee extract with those in dietary supplements. A gradient elution mode was utilized to analyze CGA and caffeine alongside various other compounds. Ultimately, the study reported that the quality of the analyzed dietary supplements was unsatisfactory [57].

#### 2.2.5. High-Performance Liquid Chromatography-Tandem Mass Spectrometry (HPLC-MS/MS)

The significance of HPLC-MS/MS lies in its unparalleled sensitivity, specificity, and versatility, making it indispensable for analyzing complex biological, environmental, and pharmaceutical compounds. This technology was used to characterize bioactive compounds in coffee silverskin. Notably, their method successfully employed polarity switching within a single chromatographic run while maintaining high analytical stability [29].

An HPLC-ESI-MS-MS approach was developed to quantify polyphenols in human urine following the consumption of various polyphenol-rich beverages, including coffee, cocoa, tea, fruit juice, wine, cider, and beer. The total run time was optimized to 6 min per sample, specifically to facilitate the high-throughput demands of epidemiological research. Peaks were identified by comparing the retention times and mass spectral characteristics of the samples against known analytical standards [79].

#### 2.2.6. High-Performance Thin-Layer Chromatography (HPTLC)

HPTLC was employed to analyze ethanol–water extracts (70:30, *v*/*v*) obtained from coffee by-products via liquid-phase UAE. The separations were performed on silica gel 60. 5-CQA and caffeine were initially detected at 254 and 366 nm, respectively [36]. For a more comprehensive characterization, the same extracts were analyzed using UHPLC coupled with UV/Vis detection and TOF mass spectrometry (UHPLC-PDA/UV-ESI-QTOF-MS/MS). Ultimately, UHPLC-PDA/UV-ESI-QTOF-MS/MS was able to identify a larger number of compounds than HPTLC [36].

#### 2.2.7. Ultra-High-Performance Liquid Chromatography–Quadrupole Time-of-Flight–Mass Spectrometry (UPLC-QTOF-MS)

A targeted quantitative analysis was performed using HPLC–triple quadrupole–mass spectrometry (UPLC-TQD-MS) and UPLC-QTOF-MS to identify chemotaxonomic markers and evaluate the chemodiversity in *Ilex guayusa*, *Ilex paraguariensis*, and *Ilex vomitoria* [62]. Multiple-reaction monitoring (MRM) parameters were developed and optimized for the detection and quantification of caffeine, 5-CQA, and other secondary metabolites. Quantification was achieved using calibration curves that exhibited linearity across the concentration ranges detected within the Ilex species. To ensure high-throughput analysis, a 7 min gradient elution was conducted using UPLC-TQD-MS; conversely, a more extended 13 min gradient run was utilized to provide higher chromatographic resolution, thereby facilitating a more precise interpretation and detection of isomeric compounds [62].

#### 2.2.8. Nuclear Magnetic Resonance (NMR)

NMR spectroscopy is a fundamental analytical tool for identifying organic compounds, as it provides a comprehensive suite of 1D and 2D NMR spectral data [80]. It has been extensively utilized to analyze both roasted and unroasted coffee extracts, as well as to identify coffee species and their geographical origins [12].

^1^H NMR-based protocol was developed to analyze, characterize, and quantify coffee compounds in espresso and Moka extracts, enabling automated metabolite analysis in approximately two minutes per spectrum. While 2D NMR was utilized for component identification, the authors demonstrated that the method effectively achieved high-throughput quantification [12]. Similarly, ^1^H and ^13^C NMR spectroscopy was employed to identify the components of roasted coffee bean extracts. Their developed method provided detailed structural information on the extract in a single experiment, leading the authors to conclude that the approach is highly suitable for analyzing other complex mixtures [18].

Furthermore, NMR was utilized to qualitatively and quantitatively characterize aqueous extracts of roasted and unroasted coffee silverskin. Specifically, ^1^H NMR was used to compare the extract composition before and after roasting, while 1D and 2D NMR facilitated the identification of major components. The study found that caffeine and 5-CQA were the predominant bioactive compounds in these aqueous extracts [80]. Nemzer et al. (2022) applied ^1^H and ^13^C NMR spectroscopy for the structural analysis of caffeine-enriched whole coffee cherry extracts; their results indicated that quantification of caffeine using ^1^H NMR yielded data comparable to traditional HPLC analysis [26].

#### 2.2.9. Infrared Spectroscopy (IR)

Limited information has been reported regarding the implementation of IR in the structural identification of coffee byproducts [30]. Therefore, IR spectroscopy was used for the structural analysis of coffee silverskin bioactive compounds. Characteristic IR bands were observed at a low frequency (750–1800 cm^−1^) and high frequency (2700–3400 cm^−1^). Meanwhile, caffeine and CGA levels were quantified using capillary zone electrophoresis (CZE). Electropherograms were detected at 280 nm, and IR spectra were scanned from 190 to 600 cm^−1^. In conclusion, the applied tools successfully enabled the analysis and isolation of compounds to provide a high molecular weight fraction with in vitro antioxidant capacity [30].

#### 2.2.10. Ultraviolet/Visible Spectroscopy (UV/Vis)

Several studies have utilized UV/Vis spectroscopy to determine caffeine and CGA levels in coffee extracts. For instance, Smrke et al. (2015) employed an HPLC-MS system to analyze green coffee extracts; simultaneously, quantification of these compounds was carried out via UV/Vis detection at 325 nm for CGAs and 275 nm for caffeine [48]. Furthermore, UV/Vis analysis was implemented to quantify caffeine and CGAs in green coffee bean extracts. Both studies validated UV/Vis spectroscopy as a viable, cost-effective alternative to gel filtration chromatography and reversed-phase HPLC for the determination of these bioactive compounds [19,23].

#### 2.2.11. Other Techniques

Several other approaches have also been documented. For example, Lemos et al. (2020) developed an ESI linear ion trap ORBITRAP (ESI-LTQ-ORBITRAP) to analyze green coffee bean extract, followed by an ESI-HRMS method to quantify caffeine and phenolic acids [46]. Furthermore, HS-SPME/GC-MS was employed for the identification and quantification of volatile components in roasted coffee bean extract [65]. Similarly, volatile compounds were identified and semi-quantified using HS-SPME/GC-MS, although caffeine and CGA analyses were performed using UHPLC-DAD, with quantification relying solely on DAD detection [11]. The 124 selected studies are summarized in Table 3, which details the various extraction, identification, and determination methods utilized for caffeine and CGAs.

Current research reveals an extensive and expanding body of literature utilizing diverse solvents, instruments, and conditions alongside conventional extraction methods. Conventional techniques, such as liquid–liquid extraction (LLE), have been widely adopted due to their simplicity and established protocols; however, they often require large volumes of organic solvents, extended extraction times, and high energy consumption. As concerns regarding environmental impact and human health intensify, researchers are increasingly questioning the sustainability of these traditional approaches. Consequently, the recent literature shows a definitive shift toward green extraction techniques—including MAE, UAE, PHWE, SFE, and SPE—which eliminate toxic solvents, facilitate selective extraction under mild conditions, and enhance mass transfer to reduce both time and solvent usage. Another significant trend is the replacement of traditional organic solvents with greener alternatives, such as water, ethanol, and natural deep eutectic solvents (NADES). Because these alternatives are biodegradable, less toxic, and derived from renewable resources, they reflect a more holistic and modern approach to chemical sustainability.

### 2.3. Assessment of Heterogeneity

The heterogeneity among the selected articles was assessed by examining variability in study characteristics, methodologies, and reported outcomes. Significant heterogeneity was observed across the selected articles, primarily driven by differences in extraction techniques, solvent systems, experimental conditions, analytical methods, and outcome measures expressed in inconsistent units. Variability in sample type, sample origin, coffee species, and processing conditions likely contributed to the disparate levels of caffeine and CGAs reported. Due to the substantial heterogeneity identified across analytical, methodological, and outcome domains, statistical pooling of results was deemed inappropriate, and a meta-analysis was not feasible. Consequently, formal quantitative measures of heterogeneity were not applied, and a qualitative synthesis approach was adopted. Furthermore, the lack of standardized protocols limits direct comparability and highlights the need for harmonized methodologies in future research.

### 2.4. Risk of Bias

The risk of bias for the selected articles was assessed using a simplified domain-based approach tailored for extraction and quantification studies of caffeine and CGAs. The assessment considered sample selection and matrix, extraction procedure, analytical technique/instrumentation, method conditions and parameters, and data, calibration, and quantification. Each domain was rated as low, high, or unclear risk of bias. The overall risk of bias was determined qualitatively based on domain-level judgements. A detailed summary of the risk of bias assessment for all included studies is presented in Table 4.

The risk of bias assessment revealed that most included articles demonstrated a low risk across core analytical domains, particularly in instrumentation and extraction procedures. However, a recurring concern was the insufficient reporting of method conditions and calibration parameters, which resulted in several studies being classified as having a high or unclear risk in these domains. Some studies lacked detailed descriptions of extraction parameters or analytical calibration methods, potentially compromising reproducibility. Additionally, inconsistencies in sample preparation and matrix selection compounded the heterogeneity across studies. This lack of standardization and transparency limits reproducibility and complicates cross-study comparisons. Furthermore, the absence of a validated risk of bias tool and the lack of quantitative integration of bias represent important methodological limitations of this review.

Given the heterogeneity of study designs and the predominance of methodological and analytical research, a formal assessment of publication bias was not conducted. However, efforts were made to mitigate potential bias by including multiple databases and screening reference lists to capture a comprehensive range of relevant literature.

## 3. Materials and Methods

This systematic review was conducted according to the Preferred Reporting Items for Systematic Reviews and Meta-Analyses (PRISMA) guidelines [146].

### 3.1. Data Sources and Objective

A cohort of 124 articles was selected from three different bibliographic databases: PubMed, Science.gov, and BASE. The inclusion criteria focused on studies evaluating the extraction protocols and analytical quantification techniques for specific bioactive compounds—namely caffeine and CGAs—across diverse sample matrices.

The systematic review aimed to evaluate and synthesize the analytical methods used for the extraction and quantification of caffeine and CGAs across plant-based matrices. The focus was on studies reporting experimental methodologies and analytical performance parameters.

### 3.2. Search Strategy

A systematic literature search was conducted in PubMed, Science.gov, and BASE, covering the period from January 1977 to January 2025. The search strategy combined keywords and Boolean operators as follows: (“caffeine” OR “chlorogenic acid” OR “CGA”) AND (“extraction” OR “green extraction” OR “determination” OR “quantification”). Searches were restricted to titles and abstracts. Only peer-reviewed articles published in English were included. The final search was performed in January 2025.

### 3.3. Eligibility Criteria

Studies were considered eligible if they reported experimental methodologies, analytical techniques, or comparative evaluations of extraction and quantification approaches for caffeine and CGAs from plant-based matrices (e.g., coffee, tea, and related species). Studies were excluded if they were reviews, patents, theses, non-English publications, or if they lacked the full text or did not provide adequate experimental or analytical information. No restrictions were applied regarding publication year.

### 3.4. Study Selection Process

The study selection process was conducted independently by four reviewers. After removing duplicates, titles and abstracts were screened according to predefined eligibility criteria. Full texts of potentially relevant studies were then assessed for inclusion. Any disagreements between reviewers were resolved through discussion and consensus. In cases where consensus could not be reached, discrepancies were resolved in consultation with the corresponding author. Consequently, 4 of 126 studies were selected from Science.gov, 12 of 45 were selected from BASE, and 108 of 148 studies were selected from PubMed. The selection process is summarized in the PRISMA flow diagram (Figure 1).

### 3.5. Data Extraction and Quality Evaluation

Data extraction and analysis were performed independently by four authors using a standardized extraction form. Extracted data included study characteristics, sample types, extraction methods, analytical techniques, instrumentation, instrumental conditions, and key findings. To ensure consistency and accuracy, the extracted data were cross-checked among the reviewers. Any discrepancies were resolved through discussion and verification against the original articles or by consulting the corresponding author.

The primary data items extracted from the included studies comprised (i) the analyte, (ii) the plant matrix or sample source, (iii) the extraction method and conditions, (iv) analytical and quantification techniques (e.g., HPLC, LC-MS, UV/Vis, UPLC), and (v) instrumentation parameters.

### 3.6. Risk of Bias Assessment

The methodological quality and risk of bias of the included studies were assessed using a modified critical appraisal approach adapted for analytical and experimental studies. The assessment focused on factors such as the clarity of methodology, the rigor of the extraction method, the validation of analytical techniques, and the completeness of the reported data. Studies were not excluded based solely on quality assessment; however, potential sources of bias were considered during data interpretation.

The risk of bias was evaluated using several domains: sample selection and matrix type, extraction procedure, analytical technique/instrumentation, method conditions and parameters, and data, calibration, and quantification. The results for these domains were graded as a low, high, or unclear risk of bias.

## 4. Conclusions

In accordance with the PRISMA guidelines, this systematic review comprehensively evaluated published articles on the extraction and determination of caffeine and CGAs in several matrices. Synthesizing a total of 124 studies published between January 1977 and January 2025, this review details a wide array of extraction techniques—ranging from conventional solvent-based methods to advanced green approaches, such as SPE, UAE, MAE, SC-CO_2_, and PHWE, each offering distinct advantages in terms of efficiency, selectivity, and sensitivity. This review bridges gaps in the existing literature left by previous reviews by integrating both conventional and green extraction methods, with a dual focus on extraction and quantification. Furthermore, quantification methods have progressed from basic spectrophotometric techniques to highly sensitive and selective chromatographic approaches, specifically HPLC and UHPLC coupled with UV or mass spectrometric detection. Overall, the findings highlight ongoing advancements toward improved efficiency and sensitivity in analytical workflows, alongside increasing interest in greener and faster extraction techniques.

## 5. Study Limitations

This systematic review is subject to several limitations. First, the heterogeneity of the included studies in terms of extraction procedures and analytical techniques limits the feasibility of direct comparisons between results. Second, the reliance on a limited set of databases (PubMed, BASE, and Science.gov) and simplified keyword combinations creates several issues such as database bias and keyword sensitivity. Third, the lack of risk of bias assessments and standardized tools—such as GRADE or Cochrane RoB—may diminish confidence in comparative claims. Fourth, the lack of standardized reporting across studies may affect reproducibility and interpretation. Fifth, the exclusion of non-English publications may introduce language bias. Finally, the absence of a formal quantitative synthesis or meta-analysis restricts the ability to provide statistically robust conclusions. The variability in sample matrices could also affect the analyte stability and extraction efficiency of caffeine and CGAs, since matrix impacts are rarely addressed or discussed.

## Figures and Tables

**Table 1:** Comparative analysis between caffeine and CGAs.

Feature	Caffeine	CGAs
Chemical Class	Alkaloid (methylxanthine)	Polyphenol (phenolic acid & hydroxycinnamate ester)
Chemical Structure	1,3,7-trimethylxanthine	Ester of caffeic acid and quinic acid
Molecular Formula	C_8_H_10_N_4_O_2_	C_16_H_18_O_9_
Natural Source	Coffee, tea, cocoa	Coffee (especially green beans), fruits, vegetables
Mechanism of Action	Increase alertness (Antagonizes adenosine receptors in the CNS)	Modulates oxidative stress, inflammation, and metabolic pathways
Physiological Effects	Increased alertness, reduced fatigue, enhanced cognition	Antioxidant, anti-inflammatory, improves glucose and lipid metabolism
Neurological Effects	CNS stimulation & enhanced neurotransmission	Neuroprotective effects
Effect on the Cardiovascular System	Can increase heart rate and blood pressure	May improve vascular function and reduce risk factors
Stability (During the Roasting Process)	Relatively stable	Degrades significantly during roasting
Health Benefits	Improved alertness, possible reduced risk of some diseases	Anti-diabetic, anti-obesity, antioxidant, cardioprotective
Adverse Effects	Insomnia, anxiety, tachycardia	Safe
Functional Use	Stimulants, beverages, pharmaceuticals → increased alertness	Nutraceuticals, functional foods, supplements

**Table 2:** The major characteristics of green extraction techniques.

Technique	Principle/Mechanism	Conditions	Advantages	Limitations
UAE	Acoustic Cavitation	Low–moderate temperature, atmospheric pressure, ultrasonic frequencies	Reduced solvent use and time, Energy efficient, Improved yield	Possible degradation of sensitive compounds due to free radicals
MAE	Microwave heating causes rapid internal heating	Moderate–high temperature, short time, controlled pressure	Fast extraction, lower solvent consumption, high efficiency	Risk of thermal degradation, limited selectivity
SFE	Using fluids (e.g., CO_2_) above critical temperature/pressure	Moderate temperature, high pressure	Non-toxic solvent (e.g., CO_2_), solvent-free extracts	High cost, complex operation, limited polarity range
PHWE	Using water at high temperatures and pressure	High temperature, high pressure	Using water instead of organic solvents	Possible degradation of thermolabile compounds, requires high energy
DES	Uses eutectic mixtures (HBD + HBA) forming green solvents with tunable properties	Moderate temperature, atmospheric pressure	Biodegradable, low-toxicity solvents, high solubilization capacity	High viscosity (mass transfer issues), difficult solvent recovery

UAE: Ultrasound-assisted extraction; MAE: Microwave-assisted extraction; SFE: Supercritical fluid extraction; PHWE: Pressurized hot water extraction; DES: Deep eutectic solvents.

**Table 3:** Summary of extraction, identification, method parameters, and levels of caffeine and CGAs.

Analyte	Type of Sample	Extraction Method	Instrumentation	Column	Mobile Phase	Diluent	Lambda	Flow Rate	Contents/Yield%	Reference
Caffeine CGAs	Green coffee bean extract	LLE: 70% ethanol	HPLC-DAD	Capcellpack C18	2 mM PhA: CH_3_CN (gradient)	----	----	1.0 mL/min	10% 27%	[81]
Caffeine CGAs	Coffee leaf	LLE: Hot water	HPLC-DAD	Phenomenex Kinetex C18	0.1%TFA/H_2_O: ACN (gradient)	----	280 nm 330 nm	1.5 mL/min	0.3–95.2% 0.3–49.7%	[82]
Caffeine CGAs	Coffee pulp	LLE: Hot water	HPLC-DAD	Zorbax Eclipse XDB-C18	2%AA/water: MeOH (isocratic)	----	280 nm 327 nm	0.6 mL/min	3.82 mg/g 13.38 mg/g	[83]
Caffeine CGAs	Ground coffee	LLE: Hot water	HPLC-DAD	Supelco LC-18	H_2_O: MeOH (60:40%) (isocratic) 0.1%FA/H_2_O:ACN (85:15%) (isocratic)	M.P. MeOH	273 nm 330 nm	1.0 mL/min	131.26–150.39 µg/mL 70.66–357.85 µg/mL	[84]
Caffeine CGAs (isomers)	Spent coffee grounds	LLE: 70% ethanol	HPLC-DAD	Phenomenex Luna C-18	0.2%FA/H_2_O: FA/ACN (gradient)	70% ethanol	276 nm 325 nm	0.8 mL/min	41.58 µg/mg 1.11–4.16 µg/mg	[85]
Caffeine CGAs	Coffee charcoal	LLE: Hot water	HPLC-PDA HPLC-MS	Nucleodur 100-5-C18	0.1%MeOH: 0.1%FA/H_2_O (gradient)	----	218, 272 nm 244, 325 nm	1.0 mL/min	N/A	[86]
Caffeine CGAs	Coffee beans	LLE: Hot water	HPLC-DAD	Agilent C-18	0.2%PhA: ACN (90:10%) (isocratic)	----	275 nm 330 nm	1.0 mL/min	0.53–49.58 µg/mg 5.15–10.61 µg/mg	[87]
Caffeine CGAs	Green coffee beans	----	HPLC-DAD-MS	Agilent Eclipse XDB-C18	ACN: 0.1%FA/H_2_O (isocratic)	----	254 nm 330 nm	0.125 mL/min	0.593% (*w*/*w*) 0.308–0.520% (*w*/*w*)	[88]
Caffeine CGAs	Coffee pulp	LLE: Hot water	HPLC-DAD	Eclipse XDB-C18	2%AA/H_2_O: MeOH (isocratic)	----	280 nm	0.5 mL/min	3.55 mg/g 12.04 mg/g	[89]
Caffeine CGAs	Mate (*Ilex paraguariensis*)	LLE: Hot water	HPLC-PDA	Phenomenex C18	0.2%AA/H_2_O: 0.2%AA/MeOH (gradient)	----	----	0.8 mL/min	N/A	[90]
Caffeine CGAs	Green & roasted coffee beans	LLE: M.P. 20%ACN/H_2_O	HPLC-PDA	Hypersil C-18 BDS	10%ACN: 0.5%AA/H_2_O (isocratic) MeOH: 0.1%FA/H_2_O (5:95%) (gradient)	M.P. 0.5% FA/H_2_O	280 nm 325 nm	0.5 mL/min 1.0 mL/min	1.0–1.8 g/100 g 2.84 g/100 g	[91]
Caffeine CGAs	Coffee beans	LLE: Hot water	UHPLC-ESI-MS	Kinetex EVO C18 Corre-Shell	0.1%FA/H_2_O: 0.1%FA/ACN (gradient)	----	280 nm 320 nm	0.2 mL/min	3.91–6.11 g/100 g 0.06–9.67 g/100 g	[92]
Caffeine CGAs	Coffee beans	LLE: Hot water	HPLC-MS/MS	Gemini C18 Polar-RP 80	0.3%FA/H_2_O: MeOH (gradient) 0.1%FA/H_2_O: 0.1%FA/MeOH (gradient)	MeOH	270 nm 325 nm	0.4 mL/min 1.0 mL/min	23,740–26,520 mg/kg 700–24,940 mg/kg	[93]
Caffeine CGAs	Coffee beans	LLE: Hot water	UHPLC–ESI–MS	Kinetex EVO C18 Corre-Shell	0.1%FA/H_2_O: 0.1%FA/ACN (gradient)	----	280 nm 320 nm	0.20 mL/min	10.0–21.9 g/100 g 3.09–50.41 g/100 g	[94]
Caffeine CGAs	Coffee cherries	LLE: Hot water	HPLC-DAD	Kinetex, Biphenyl	1%AA: MeOH (20:80%) (isocratic)	----	273 nm	1.0 mL/min	11.9–13.0 mg/g 5.4–10.8 mg/g	[95]
Caffeine CGAs	Coffee beans	LLE: Hot water	UV/Vis HPLC-UV LC-MS	SunFire C18 Symmetry C18	H_2_O: ACN (gradient) H_2_O: MeOH (gradient)	----	272 nm 325 nm	1.0 mL/min	N/A	[96]
Caffeine CGAs	Coffee beans	LLE: Hot water	HPLC-DAD	Agilent Eclipse XDB C18	2%AA/H_2_O: ACN (gradient)	----	254 nm (caffeine)	0.80 mL/min	9.880–11.868 mg/g 2.584–34.181 mg/g	[97]
Caffeine CGAs	Coffee beans	LLE: Hot water	UHPLC-DAD	Accucore C18	1%FA/H_2_O: 1%FA/ACN (gradient)	----	280 nm 320 nm	0.50 mL/min	1352.6–1977.9 mg/100 g 2960.0–6415.0 mg/100 g	[98]
Caffeine CGAs	Coffee beans	----	HPLC-UV/Vis HPLC-DAD	Lichrosorb 100 RP-18 Spherisorb S5 ODS2	Ph. Buffer: ACN (90:10%) (isocratic) Citrate Buffer: MeOH (gradient)	----	254 nm 325, 330 nm	1.0 mL/min 1.0 mL/min	1.127–2.163 mg/cm^3^ 0.017–3.997 mg/cm^3^	[99]
Caffeine CGAs	Coffee beans	LLE: MeOH: H_2_O (20:80%)	HPLC-DAD	Lichrosphere C18	ACN: Ph. Buffer (pH = 2.7) (gradient)	MeOH: H_2_O	272 nm 324 nm	1.0 mL/min	1110–1884 mg/100 g 234–4946 mg/100 g	[100]
Caffeine CGAs	Coffee beans	----	HPLC	SP column C18	0.1%AA/H_2_O: ACN (gradient)	----	----	1.0 mL/min	85.696–92.032 µg/g 0.227–5.735 µg/g	[101]
Caffeine CGAs	Coffee beans	LLE: Hot water	HPLC-DAD	Supelcosil LC-18-DB	H_2_O: MeOH (60:40%) (isocratic) 0.1% FA/H_2_O: ACN (85:15%) (isocratic)	M.P. MeOH	274 nm 330 nm	1.0 mL/min	166.72–203.63 mg/L 90.53–543.23 mg/L	[102]
Caffeine CGAs	Coffee beans	SPE: 70% MeOH	UHPLC-DAD	XDB-C18	ACN: 0.5% AA/H_2_O (gradient)	MeOH	276 nm 325 nm	0.3 mL/min	11.8–11.9 g/kg.dw 1.0–11.8 g/kg.dw	[103]
Caffeine CGAs	Coffee beans	LLE: Hot water	HPLC-DAD	Supelco C18	95% 2.0 mM PhA/5% MeOH: 95% MeOH/5% 2.0 mM PhA (75:25%) (isocratic)	----	280 nm 325 nm	1.0 mL/min	930–1230 mg/L 330–520 mg/L	[104]
Caffeine CGAs	Chinese Tea	LLE: THF:MeOH:AA/H_2_O (50:3.7:46.3), and DEE-EA (1:1)	HPLC-PDA	Agilent Zorbax Extend-C18	0.1%FA/H_2_O: MeOH (gradient)	----	----	1.0 mL/min	12.273–41.631 mg/g.dw 0.176–0.374 mg/g.dw	[72]
Caffeine CGAs	Coffee beans	LLE: Hot water	HPLC-DAD	Gemini C18	0.1%FA/H_2_O:MeOH: H_2_O (gradient)	----	270 nm 325 nm	0.8 mL/min	727.26–2903.14 mg/L 30.77–3011.89 mg/L	[47]
Caffeine CGAs	Coffee beans	LLE: Hot water	HPLC-PDA	DS Hypersil C18	0.2% o-PhA: ACN (90:10%) (isocratic)	Water	275 nm 323 nm	1.0 mL/min	1.89–3.05 g/100 g 0.60–2.32 g/100 g	[105]
Caffeine CGAs	Coffee beans	LLE: Cold and hot water	HPLC	Kinetex EVO C-18 column	0.05% PhA/H_2_O (isocratic)	Water	210 nm	0.5–0.8 mL/min	47.3–120.4 mg/100 mL 8.12–27.2 mg/100 mL	[106]
Caffeine CGAs	Coffee cherry pulp	Soxhlet extraction: 95% ethanol	HPLC-UV	RP C18	ACN: 1% AA (15:85%) (isocratic)	MeOH	280 nm	1.0 mL/min	26.8–45.0 mg/g 6.2–17.3 mg/g	[28]
Caffeine CGAs	Mate (*Ilex paraguariensis* A. St.-Hil.)	LLE: Water (pH = 6)	HPLC-UV	Shim-pack C18	ACN: 0.1% FA (15:85%) (isocratic) H_2_O: AA: *n*-butanol (350:1:10 *v*/*v*/*v*) (isocratic)	Water	272 nm 280 nm	1.0 mL/min 0.8 mL/min	1253–3142 µg/mL 1862–9004 µg/mL	[107]
Caffeine CGAs	Coffee silverskin	LLE: Hot water	CE-UV	----	----	----	200 nm 280 nm 420 nm	----	3.02–7.70 g/100 g 751.2–1053.8 mg/100 g	[108]
CGAs	Hydrocotyle sibthorpioides	LLE: Water (pH = 2) & EA	UHPLC–MS/MS	Hypersil Gold C18	10 mM AAc/H_2_O (pH 5.0 with 0.01%FA): ACN (gradient)	----	270 nm	0.5 mL/ min	119.4–180.8 mg/kg.dw	[73]
Caffeine CGAs	Coffee cherry pulp	LLE: Hot water	HPLC-DAD	Kinetex EVO C18	1% FA/H_2_O: 1%FA/ACN (gradient)	Water	260 nm 280 nm 320 nm 340 nm	----	226.4 mg/L 69.6 mg/L	[109]
Caffeine CGAs	Coffee silverskin	LLE: Hydroalcoholic solvent	HPLC-DAD	Tracer-Excel ODSA	0.5% AA/H_2_O: MeOH (gradient)	Hydroalcoholic solvent	274 nm 320 nm	0.7 mL/min	1.25 g/100 g 246 mg/100 g	[110]
Caffeine CGAs	Green coffee seeds	LLE: MeOH: EtOH	UPLC-ESI-QTOF-MS	HSS C18 SB Waters	2%AA/H_2_O: 0.5% AA/ACN (50:50%) (gradient)	MeOH: H_2_O (90:10)	280 nm	0.55 mL/min	5.9–7.2% 13.2–27.9%	[111]
Caffeine CGAs	Conilon and Arabica coffee flowers	LLE: Hot water 70% EtOH	HPLC-UV	RP C18	30% MeOH: 1% AA (isocratic)	Water 70% EtOH	274 nm 325 nm	1.4 mL/min	498.7–3699.3 mg/100 g 7.0–53.0 mg/100 g	[25]
Caffeine CGAs	Coffee pulp	LLE: Distilled water DCM	UV-Vis Absorption Spectroscopy	----	----	DCM H_2_O	274 nm 324 nm	----	0.08–0.16 g/100 g.dw 0.83–1.22 g/100 g.dw	[112]
Caffeine CGAs	Coffee beans & coffee by-products	LLE: Hot water	LC-ESI-QTOF-MS	C18	15% MeOH: 85% MeOH: H_2_O [30:70]—2%AA (pH 3.4) (gradient)	0.1% FA/H_2_O: 0.1% FA/ACN	280 nm 320 nm	0.5 mL/min	13.08–26.80 mg/g 1.99–13.45 mg/g	[113]
Caffeine CGAs	Selected medicinal plants	LLE: 80% MeOH	UHPLC-MS	Gemini C18	0.1% FA/H_2_O: 0.1% FA/ACN (isocratic)	MeOH: H_2_O (50:50%)	----	0.2 mL/min	11.23–564.45 mg/L 199.03–783.80 mg/L	[114]
Caffeine CGAs	Coffee silverskin	LLE: Hot water	UHPLC-PDA-ESI-TOF/MS	Waters Acquity BEH C18	0.02% FA/H_2_O: 0.02% FA/ACN (95:5%) (gradient)	MeOH	275 nm 324 nm	0.6 mL/min	2.821–15.132 mg/g 0.202–72.938 mg/g	[115]
Caffeine CGAs	Coffee leaves	Soxhlet extraction: *n*-Hexane & MeOH	HPLC-DAD HPLC-ESI-MS	Gemini C18 Zorbax Eclipse Plus C18	2% AA/H_2_O: ACN (gradient)	----	278 nm 320 nm	1.0 mL/min	5.87–9.68 g/kg 2.07–17.51 g/kg	[116]
Caffeine CGAs	Coffee silverskin	LLE: Water, MeOH, EtOH	HPLC-MS/MS	Kinetex PFP	0.1% FA/H_2_O: 0.1% FA/MeOH (gradient)	----	----	0.2 mL/min	10.010–35.879 mg/g 0.0437–4.014 mg/g	[117]
CGAs	Sunflower hulls	Soxhlet extraction: *n*-Hexane	HPLC-DAD	----	MeOH: 2%AA/H_2_O (gradient)	----	328 nm	1.0 mL/min	601.82–654.89 mg/100 g	[71]
Caffeine CGAs	Green coffee	LLE: Hot water	UPLC	RP-C18	MeOH: H_2_O: AA (20:80:1%) (isocratic)	Water	272 nm	0.1 mL/min	9.22–9.67 mg/g 17.79–18.71 mg/g	[118]
Caffeine CGAs	Coffee residues	LLE: H_2_O, MeOH, EtOH, and *n*-Hexane	HPLC-PDA	Lichrosorb RP 18	50 mM Ph. Buffer, (pH = 2.6): 0.2 mM *o*-PhA (pH 1.5): 20% Buffer/80% ACN (gradient)	----	280 nm	1.0 mL/min	0.640–8.370 mg/mL 0.490–5.780 mg/mL	[119]
CGAs	Brewed coffee	LLE: H_2_O & Dichloromethane	HPLC	Zorbax Eclipse XDB C-18	10 mM CA: MeOH (gradient)	----	325 nm	1.0 mL/min	15.5–1032.6 µg/mL	[70]
CGAs	Green tea *Camellia crassicolumna Var. multiplex*	LLE: H_2_O, Acetone, Chloroform, EA	HPLC-DAD	Zorbax SB-C18	0.34% PhA/H_2_O: ACN (gradient)	----	280 nm	1.0 mL/min	N/A	[120]
Caffeine CGAs	Coffee fruit	LLE: EtOH, H_2_O	HPLC-DAD HPLC-PDA-MS	Supelco Phenyl-Hexyl RP C18 Phenomenex	0.1% PA: ACN (90:10%) (isocratic) 1.0% FA/ACN (gradient)	MP 50% EtOH/H_2_O	275 nm 325 nm	0.8 mL/min 0.3 mL/min	0.44–1.03% 0.3–376.7%	[121]
Caffeine CGAs	Spent coffee grounds	LLE: 50% EtOH/H_2_O	UHPLC-PDA-TOF-MS	Waters Acquity BEH C18	0.02% FA/H_2_O: 0.02% FA/ACN (gradient)	MeOH	210–500 nm	0.6 mL/min	0.96–11.5 mg/g 1.65–6.09 mg/g	[122]
Caffeine CGAs	Coffee cherries	LLE: 70% ethanol/water	LC-MS/MS	Agilent Poroshell 120 EC-C18	0.1% FA/H_2_O: 0.1% FA/ACN (gradient)	50% MeOH/H_2_O Water	----	0.4 mL/min	70% 40%	[123]
Caffeine CGAs	Coffee beans	LLE: MeOH: H_2_O (70:30%)	HPLC-UV	Phenomenex Gemini C-18	0.1% FA/H_2_O: 0.1% FA/ACN (gradient)	----	276 nm 325 nm	0.8 mL/min	18.69–26.28 mg/g 1.68–78.33 mg/g	[124]
Caffeine CGAs	Coffee beans and silverskin	LLE: 70% EtOH/water	HPLC-UV LC/UV/ESI-MS	Kinetex C-18	0.1% FA/H_2_O: 0.1% FA/ACN (gradient)	70% EtOH/H_2_O	275 nm 330 nm	0.35 mL/min	0.98–104.28 mg/g.dw 27.83–48.13% (*w*/*w*)	[125]
Caffeine CGAs	Brewed coffee	LLE: Water	HPLC-DAD	SCR-102H	2% AA/H_2_O: 0.5% AA/50% ACN (gradient)	----	278 nm 320 nm	1.0 mL/min	1.70–4.98 mg/g 3.08–10.65 mg/g	[126]
Caffeine CGAs	Mate *Ilex paraguariensis*	LLE: Hot water	HPLC-PDA HPLC-DAD-ESI/MS	X-Bridge C18	1%FA/H_2_O: 1%FA/ACN (gradient)	Water	272 nm 326 nm	1.0 mL/min	114–268 µg/mL 102–289 µg/mL	[78]
Caffeine CGAs	Coffee cherries	LLE: 70% EtOH/water 50% MeOH/H_2_O	HPLC-PDA	Supelco Phenyl Hexyl Phenomenex Luna C18	0.1% PA/H_2_O (90:10%) 2% AA/H_2_O: ACN (gradient)	50% MeOH/H_2_O	275 nm 325 nm	0.6 mL/min 1.0 mL/min	0.90–73.60% (*w*/*w*) 6.10–46.46% (*w*/*w*)	[127]
Caffeine CGAs	Coffee bean bagasse	LLE: Ethanol/water	HPLC-DAD	Supelcosil LC-18	5% FA/H_2_O: MeOH (gradient)	----	260, 280, 320, 330, & 360 nm	1.5 mL/min	5526.55–12,528.31 µg/g 129.33–227.27 µg/g	[37]
Caffeine CGAs	Coffee bean & cherries	LLE: Hot water	HPLC-PDA	Supelco RP-18	0.5% TFA/H_2_O: ACN (gradient)	----	280 nm 330 nm	0.9 mL/min	0.87–3.88 mg/g 14.52–55.75 mg/g	[128]
Caffeine CGAs	Kombucha tea	LLE: Water	HPLC-UV	Eurospher (100-5) C-18	1% AA/ACN: MeOH (10:90%) (isocratic)	----	----	1.0 mL/min	102.87–165.49 mg/dm^3^ 5.13–7.60 mg/dm^3^	[129]
Caffeine CGA	Green coffee beans	LLE: 50% EtOH/water	HPLC LC-MS	Kinetex RP-C18	0.1% FA/H_2_O: 0.1% FA/ACN (gradient)	----	----	0.2 mL/min	0.79–1.84 g/kg 0.19–0.49 g/kg	[130]
Caffeine CGAs	Coffee cherries	LLE: Water	HPLC-PDA	Kinetex XB C18	0.1% FA/H_2_O: MeOH (gradient)	----	272 nm 325 nm	1.0 mL/min	601–795 mg/100 g 227–897 mg/100 g	[131]
Caffeine CGAs	Spent coffee grounds and coffee husks	Supercritical fluid, ultrasound, and Soxhlet extraction (Hexane, DCM, EA, and EtOH)	RP-HPLC	Shim-pack C18	0.1% FA: ACN (85:15%) (isocratic)	----	----	0.8 mL/min	0.734–684.2 µg/mg 0.30–942.8 µg/g	[64]
CGAs isomers	Coffee leaves	LLE: Water	LC-EC LC-QTOF-MS	Atlantis C18 Poroshell 120 EC-C18	0.1 M Ph. buffer (pH 3.5): 15% MeOH (isocratic) 0.025% TFA + 0.075% FA/H_2_O: 0.025% TFA + 0.075% FA/ACN (gradient)	MeOH	----	0.8 mL/min	1.8–203 mg/L	[20]
Caffeine CGAs	Coffee bean	LLE: Hot water	HPLC LC-MS	Develosil C30 UG5	0.1% FA/H_2_O: 0.1% FA/MeOH (gradient)	----	275 nm 330 nm	1.0 mL/min 0.5 mL/min	0.144–6.775 mmol/L 0.40–1.381 mmol/L	[21]
Caffeine CGAs	Decaffeinated coffee, green tea, cocoa powder, grape skin, and grapefruit and orange juices	LLE: Hot water, EA, MeOH	HPLC-ESI-MS/MS	Zorbax Eclipse XDB-C18	0.1% FA/H_2_O: 0.1% FA/ACN (95:5%) (gradient)	Blank urine	----	0.8 mL/min	2.1–14.1 mg/g 1.6–20.6 mg/g	[79]
Caffeine CGAs	Coffee and willow	LLE: Hot water	UPLC	BEH C18	0.1% FA/H_2_O: 0.1% FA/MeCN (gradient)	Water	220 nm to 480 nm	0.4 mL/min	15.3 mg/g ND	[53]
Caffeine CGAs	Coffee Refuse	LLE: Hot water, EA	HPLC-PDA	Inertsil ODS-SP	MeOH: 5 mM Ph. Buffer (pH = 2.5) (30:70%) (isocratic)	----	280 nm	0.4 mL/min	173.0 µg/mL 62.3 µg/mL	[34]
Caffeine CGAs	Green coffee bean	LLE: ACN, Water	LC-MS/MS HPLC-UV	Synergi Polar-RP	----	----	----	----	60.0 mg/s 6.84–11.4 ng/mL	[5]
Caffeine CGAs	Green coffee bean	SPE: Water, MeOH (70:30%)	HPLC-DAD	Kinetex C18	0.1% FA/H_2_O: 0.1% FA/MeOH (gradient)	MeOH/water	250 nm to 400 nm	1.0 mL/min	7.31–61.53 mg/g 25.7–152.1 mg/g	[16]
Caffeine CGAs	Coffee silverskin	LLE: Hot water	UV-Vis Absorption Spectroscopy CE-DAD	----	0.1 M NaOH and borate buffer	----	280 nm	----	0.1–3.5% (*w*/*w*) 0.2–1.2% (*w*/*w*)	[30]
Caffeine CGAs	Brewed coffee	LLE: Water	HPLC-UV	XBridge. Shield RP18	10 mM CA/H_2_O: MeOH (gradient)	----	276 nm 325 nm	1.0 mL/min	0.59–1.05 mg/mL 0.10–0.25 mg/mL	[45]
Caffeine CGAs	Brewed coffee	LLE: Hot water	HPLC-DAD	Poroshell 120, ECC18	FA/H_2_O: ACN/H_2_O (pH 3.2) (gradient)	Water	270 nm 330 nm	0.4 mL/min	1.32–2.57 mg/mL 1.95–3.99 mg/mL	[56]
Caffeine CGAs	Coffee husks	LLE: MeOH/H_2_O (50:50%) and acetone/H_2_O (70:30%)	HPLC-PDA	Shimadzu C18	H_2_O/ACN (92.6:7), 0.4% PhA: ACN, 0.4% PhA (gradient)	Water	272 nm 325 nm	1.2 mL/min	418.13–696.22 mg/100 g 17.19–174.27 mg/100 g	[32]
Caffeine CGAs	Ziyang green tea	LLE: 50% EtOH/water 35% MeOH/water	HPLC-UV	Agilent Zorbax SB-C18	0.1% TFA/MeOH (95:5%, pH 2.28): MeOH (gradient)	MeOH	278 nm	1.0 mL/min	26.71–38.06 mg/g 0.19–1.91 mg/g	[39]
Caffeine CGAs	Green coffee (Catuai and Tipica)	LLE: Hot water Soxhlet; MeOH	HPLC-UV HPLC-MS HPSEC	Poroshell 120 EC-C18	MeOH/H_2_O (10:90%) with 0.1% FA: MeOH/H_2_O (95:5%) with 0.1% FA (gradient)	MeOH	275 nm 325 nm	0.3 mL/min	0.97–1.07% (*w*/*w*) 4.10–5.41% (*w*/*w*)	[48]
CGAs	Fermentation broth and fruits (Mango)	LLE: EtOH, MeOH, Water	UV-Vis Absorption Spectroscopy HPLC-UV/Vis	JADE-PAK ODS-AQ C18	0.5% AA: ACN (90:10%) (isocratic)	EtOH, MeOH, and DMSO	327 nm	1.0 mL/min	0.48–23.56 mg/L 0.46–23.39 mg/L	[74]
Caffeine CGAs	Coffee flower	SLE, PHWE, and Soxhlet: EtOH, Water	HPLC-UV	Reversed-phase SS-C18	1% AA/ACN: 1% AA/H_2_O (gradient)	----	270 nm 320 nm	0.4 mL/min	1070.8 mg/100 g ND	[24]
Caffeine CGAs	Coffee beans	LLE: Hot water, MeOH	NMR	----	----	Water	----	----	28.38–91.92 µg/mg 86.51–349.59 µg/mg	[12]
Caffeine CGAs	Coffee beans	LLE: Hot water	HPLC-VWD	Gemini C18 Polar-RP 80	0.3% FA/H_2_O: MeOH (gradient) 0.1% FA/H_2_O: 0.1% FA/MeOH (gradient)	MP	270 nm 345 nm	0.4 mL/min 1.0 mL/min	33.02–174.03 mg/g 14.95–71.54 mg/g	[13]
Caffeine CGAs	Coffee beans	LLE: MeOH	ESI-HRMS	----	----	----	----	3.0 μL/min	7.7–48.5 µmol/L 4.02–18.65 µmol/L	[46]
Caffeine CGAs	Coffee beans	LLE: MeOH (BHT & AA (85:15%))	HPLC-PDA	Thermo BDS	0.15% PA/H_2_O: ACN (gradient)	MeOH (BHT & AA)	270 nm 345 nm	0.7 mL/min	9.65–15.16 mg/g 3.77–13.80 mg/g	[132]
Caffeine CGAs	Coffee by-products	LLE: EtOH: H_2_O, FA	UHPLC-PDA/UV	Phenyl-Hexyl	0.3% FA/H_2_O: EtOH/ACN (1:1) (gradient)	----	210 nm to 400 nm	0.25 mL/min	0.17–52.97 mg/g 0.59–72.94 mg/g	[36]
Caffeine CGAs	*Ilex paraguariensis* (Yerba Mate)	LLE: Hot water	HPLC-PDA UV-Vis Absorption Spectroscopy	IB-SIL RP 18	AA/H_2_O (2:98%): AA/MeOH (2:98%) (gradient)	----	270 nm 325 nm	1.0 mL/min	0.70–1.06 g/100 g 1.98–2.80 g/100 g	[42]
Caffeine CGAs	Green coffee	LLE: Water, EtOH, MeOH, Acetone	HPLC-CAD	Hypersil Gold	0.1%FA/22%ACN: 0.1%FA/78% H_2_O (isocratic)	EtOH/H_2_O (40:60%)	----	0.7 mL/min	4.13–126 mg/g 0.33–329 mg/g	[57]
Caffeine CGAs	Green coffee beans	LLE: MeOH: H_2_O (50:50%)	HPLC-PDA	Synergi Polar-RP	1%FA: 5–8% ACN/H_2_O (gradient)	MeOH: H_2_O (50:50%)	280 nm 325 nm	1.0 mL/min	12–13 mg/g 5–41 mg/g	[76]
Caffeine CGAs	Coffee beans	LLE & Ultrasound-Assisted Extraction: Hot water, cold water,	HPLC	Diamosil C18	0.1% PhA/H_2_O: ACN (90:10%) (gradient)	Water	272 nm 327 nm	1.0 mL/min	0.56–0.68 mg/mL 1.25–1.53 mg/mL	[65]
Caffeine CGAs	Brewed coffee	LLE: Hot water	HPLC-DAD	Poroshell 120, EC-C18	FA/H_2_O (pH 3.2): ACN (gradient)	Water	220 nm to 600 nm	0.4 mL/min	0.52–4.20 mg/mL 0.02–4.80 mg/mL	[58]
Caffeine CGAs	Coffee beans	MAE & SLE: Hot water	HPLC-DAD	C18	5% FA: MeOH (isocratic)	Water	280 nm 325 nm	0.8 mL/min	3.7–7.3% (*w*/*w*) 1.1–2.1% (*w*/*w*)	[63]
CGAs isomers	Coffee capsule	LLE: Hot water	HPLC-PDA	COSMOSIL C18	H_2_O/ACN/TFA (94.9:5:0.1%): ACN/TFA (99.9/0.1%) (gradient)	----	280 nm 320 nm	1.0 mL/min	0.47–2.79 mg/mL 0.26–2.42 mg/mL	[75]
Caffeine CGAs	Coffee beans	LLE: Water	UV-Vis Absorption Spectroscopy	----	----	----	256 nm 292 nm	----	1.20–1.46% 4.07–4.43%	[60]
Caffeine CGAs	Green coffee beans	LLE: 1% AA/H_2_O, ACN	RP-HPLC- DAD UV-Vis Absorption Spectroscopy	Supelco C8	0.1% o-PhA: ACN (90:10%) (isocratic)	AA/H_2_O, ACN	272 nm 325 nm	0.4 mL/min	1.01–1.31% (*w*/*w*) 8.50–8.92% (*w*/*w*)	[23]
Caffeine CGAs	Green coffee beans	LLE: 70% EtOH/water	HPLC-UV/Vis	Insertsil ODS	0.1% AA/H_2_O: ACN (gradient)	EtOH/water	272 nm 326 nm	1.0 mL/min	1.70–4.98 mg/g 3.08–10.65 mg/g	[59]
Caffeine CGAs	Spent coffee grounds	LLE: Hot water 45% EtOH/water	HPLC-DAD	Phenomenex C18	ACN: DI: FA (10:90:1.5 mL) (isocratic)	MP	280 nm	0.6 mL/min	0.537–0.583 mg/g 0.292–0.369 mg/g	[35]
Caffeine CGAs	Coffee leaf	LLE: MeOH, EtOH	LC-HRMS	Waters ACQUITY UPLC C18	0.2% FA/H_2_O: ACN (gradient)	Water MeOH	----	0.4 mL/min	24.3 mg/g 5.5–7.8 mg/g	[44]
CGA isomers	Coffee beans	LLE: 40% MeOH/Water	HPLC-DAD HPLC-MS	ODS-C18	10 mM CA/H_2_O: MeOH (gradient)	----	325 nm	0.2 mL/min	14.5–4340.0 mg/100 g	[77]
Caffeine CGAs	Brewed coffee	LLE: Hot water	RP = HPLC	XTerra MS C18	----	Water	280 nm 325 nm	1.0 mL/min	1.1–8.4% (*w*/*w*) 0.2–8.5% (*w*/*w*)	[54]
Caffeine CGAs	Spent coffee grounds	LLE: Hot water	HPLC-PDA	Phenomenex ODS-2 C18	0.01 M Ac. Buffer (pH = 3.90): MeOH (90:10%) (gradient)	----	276 nm 325 nm	1.0 mL/min	194.0–666.1 mg/100 g 227.0–706.1 mg/100 g	[27]
Caffeine CGAs	Green coffee	LLE: 20% MeOH/H_2_O	HPLC-DAD HPLC-DAD-MS	ODS-C18 Shim-pack	10 mM CA (pH 2.5)/MeOH (80:20%): MeOH (gradient)	----	272 nm 325 nm	1.0 mL/min	3923–11,776 mg/100 g 22,869–37,965 mg/100 g	[22]
Caffeine	Green coffee beans	LLE: HCl/MeOH (50:50%) Acetone/H_2_O (70:30%) 1% FA/H_2_O	LC-DAD-MS	Superspher RP18	1% FA/H_2_O: ACN: MeOH (gradient)	----	272 nm	1.0 mL/min	11.654–13.066 mg/g	[133]
Caffeine CGAs	*Ilex* leaf	LLE: 70% MeOH/H_2_O	UPLC-TQD-MS	Waters Acquity BEH C18	0.1% FA/H_2_O: ACN (gradient)	----	----	0.5 mL/min	0.004–26.94 mg/g 40.7 mg/g	[62]
Caffeine CGAs	Green coffee	LLE: 70% EtOH/H_2_O	UV/Vis Spectroscopy HPLC-PDA	Kinetex 5U C18	0.1% TFA/H_2_O: ACN (gradient)	70% EtOH/H_2_O	272 nm 330 nm	1.5 mL/min	0.85–3.65% 4.40–15.15%	[19]
Caffeine CGAs	Coffee plants leaves	LLE & SPE: MeOH, H_2_O	HPLC-SPD	Lichrosorb silica RP-18	MeOH/H_2_O: 1.0 mM HCl (gradient)	MeOH/H_2_O	272 nm 320 nm	1.0 mL/min	3.08–1486.04 ppm 240.55–922.95 ppm	[15]
Caffeine CGAs	Coffee leaves	LLE: EtOH	HPLC-PDA	Kinetex C18	0.1% TFA/H_2_O: ACN (gradient)	----	280 nm 330 nm	1.5 mL/min	5.5–12.2 mg/g 0.1–12.3 mg/g	[134]
Caffeine CGAs	Coffee beans	LLE: 5% AA, H_2_O	HPLC-UV	Spherisorb ODS1	5% AA/H_2_O: ACN (gradient)	----	272 nm 320 nm	0.7 mL/min	2.60–3.98 g/100 g 2.39–14.04 g/100 g	[135]
Caffeine CGAs isomers	Green coffee extracts	LLE: MeOH	HPLC-PDA	Agilent Zorbax RX C-18	0.1% FA/H_2_O: 0.1% FA/ACN (gradient)	MeOH	275 nm 325 nm	1.0 mL/min	0–17% 26.8–29.9%	[136]
Caffeine CGAs	Green tea, ceylon tea & mate	LLE: 70% EtOH/H_2_O H_2_O 50% MeOH/H_2_O	UHPLC-ESI-QTOF	Waters Cortecs UPLC C18	0.1% AA/MeOH: 0.1% AA/H_2_O (gradient)	50% MeOH/H_2_O	----	0.3 mL/min	1–2% 2.8%	[38]
Caffeine CGAs	Spent coffee ground	UAE & LSE: H_2_O/MeOH/EtOH	HPLC-MS/ MS	Kinetex PFP	0.1% FA/H_2_O: 0.1% FA/MeOH (gradient)	MeOH	----	0.2 mL/min	101.2–103.2 mg/kg 90.9–101.2 mg/kg	[33]
CGAs isomers	Green coffee	LLE: Hydroalcoholic solvent	HPLC-DAD	ODS-C18	10 mM CA/H_2_O (pH = 2.5)/MeOH (80:20%): MeOH (gradient)	----	325 nm	1.0 mL/min	0.03–119.8 µmol/0.4 g	[137]
Caffeine CGAs	Tea	LLE: Hot water	RP-HPLC-DAD-ESI-MS	Spherigel C18	0.5% FA/H_2_O: ACN (gradient)	Water	195 nm to 350 nm	1.0 mL/min	1.12–3.83% (*w*/*w*) 0.029–0.39% (*w*/*w*)	[138]
Caffeine CGAs	Coffee pulp, cocoa husk, and pod husk	Deep eutectic solvents: Hydrated DES/H_2_O (ChCl, Betaine, Lactic acid, Glycerol, 1,4-Butanediol)	HPLC-DAD UPLC-MS	Kinetex C18	0.1% AA/H_2_O: 0.1% AA/MeOH (gradient)	MeOH	280 nm	1.0 mL/min	0.10–0.65 g/100 g 0.18–0.55 g/100 g	[31]
Caffeine CGAs	Greek Sideritis, herbal extracts, green tea, black tea, & coffee	LLE: Hot water	HPLC-DAD	PerfectSil Target ODS-3	MeOH/ACN (95:5%): 0.1% AA/H_2_O (gradient)	MeOH/H_2_O (30:70%)	270 nm 330 nm	0.8 mL/min	101.4–321 µg/g 0.50–164.6 µg/g	[17]
Caffeine CGAs	Coffee beans	LLE: 70% EtOH/H_2_O	UPLC-DAD	Alltech Alltima C-18	0.1% AA/H_2_O: MeOH (gradient)	----	275 nm 325 nm	0.2 mL/min	1.88–2.61 g/100 g 0.75–0.87 g/100 g	[11]
Caffeine CGAs	Cocoa shell	SWE: Water	HPLC-VWD	C18	0.5% FA/H_2_O: ACN (gradient)	----	254 nm	0.89 mL/min	0.04–0.29% (*w*/*w*) 0.01–0.03% (*w*/*w*)	[43]
Caffeine CGAs	Coffee silverskin	LSE & UAE: 70% EtOH/H_2_O	HPLC-MS/MS	Kinetex PFP	0.1% FA/H_2_O: 0.1% FA/MeOH (gradient)	MeOH	----	0.2 mL/min	731.5–845.5 mg/kg 100.6–985.7 mg/kg	[29]
Caffeine CGAs	Green coffee beans	Digestion & LLE: Water DCM/H_2_O	HPLC-DAD	Enduro C18 G	MeOH: H_2_O (37:63%) (isocratic) MeOH: 1%AA/H_2_O (40:60%) (isocratic)	----	274 nm 277 nm	1.0 mL/min	0.016–1824.16 ppm 118.22–741.23 ppm	[139]
Caffeine CGAs	Coffee silverskin	LLE: D_2_O	^1^H-NMR & 2D NMR	----	----	----	----	----	N/A	[80]
Caffeine CGAs	Coffee cherry	LLE: D_2_O	^1^H, ^13^C, 2D-NOESY & 2D-HSQC NMR	----	----	----	----	----	73.25% 7.63%	[26]
Caffeine CGAs	*Ilex paraguariensis* (Yerba mate)	LLE: 80% EtOH/H_2_O Hot water	UHPLC-DAD	X-Bridge HPLC C8	0.1% FA/H_2_O (pH = 2.17): MeOH (gradient)	80% MeOH/H_2_O	280 nm	1.0 mL/min	51.16–349.19 mg/100 g 72.89–388.15 mg/100 g	[41]
Caffeine CGAs	Green coffee	LLE: Hot water	RP-HPLC-UV	Lichrosorb RP-18	20% MeOH: H_2_O (pH 4.5) (isocratic)	----	254 nm	1.5 mL/min	0.9–3.2% (*w*/*w*) 2.7–5.6% (*w*/*w*)	[14]
Caffeine CGAs	Spent coffee grounds	LLE: 50% EtOH/H_2_O	HPLC-DAD	Phenomenex Kinetex C18	0.01 M AA/Ac. Buffer (pH 3.90): MeOH (gradient)	----	272 nm 325 nm	1.0 mL/min	0.74–12.40 g/kg 157–3593 mg/kg	[140]
Caffeine CGAs	Green & Roasted Coffee	Maceration: Wine LLE: Hot Water	UHPLC-ESI-MS	Kinetex EVO C18	0.1% FA/H_2_O: 0.1% FA/ACN (gradient)	----	----	0.20 mL/min	3.41–3.52 g/100 g 0.03–5.71 g/100 g	[141]
Caffeine CGAs	*Ilex guayusa (Ilex Guayusa)* Leaves	LLE & SPE: EtOH/H_2_O	HPLC-DAD	C18 Column	0.1% FA/H_2_O: ACN (gradient)	----	330 nm 360 nm	1.0 mL/min	3.56–79.16 mg/g 5.23–43.02 mg/g	[142]
Caffeine CGAs	Coffee Pulb	SC-CO_2_: EtOH SPE: EtOH & H_2_O	UPLC-MS	XBridge BEH C18	0.01% FA/H_2_O: 0.01% FA/ACN (gradient)	EtOH: H_2_O	210–400 nm	0.70 mL/min	3.88–6.97 mg/g 6.38–58.73 µg/g	[143]
Caffeine CGAs	Wild *Camellia* species Leaves	LLE: MeOH/H_2_O	UPLC	Hypersil GOLD aQ	MeOH: 0.1% o-PhA/H_2_O (gradient)	----	265 nm	0.20 mL/min	97.43–187.37 µg/g 644.33–3233.33 µg/g	[144]
Caffeine CGAs	Coffee Beans	LLE: Hot Water	HPLC-DAD	Gemini C18	0.1% FA/H_2_O: 0.1% MeOH/H_2_O (gradient)	----	270 nm 325 nm	0.80 mL/min	72.5–290.10 mg/L 30.77–3008.76 mg/L	[145]

AA: Acetic acid, AAc: Ammonium acetate, Ac. Buffer: Acetate buffer, ACN: Acetonitrile, BHT: Butylated hydroxytoluene, CA: citric acid, CAD: Charged aerosol detector, CE: Capillary electrophoresis, DAD: Diode array detector, DCM: Dichloromethane, DEE: Diethyl ether, DI: Deionized water, DMSO: Dimethyl sulfoxide, EA: Ethyl acetate, EtOH: Ethanol, ESI: Electrospray ionization, FA: Formic acid, HPLC: High-performance liquid chromatography, HRMS: High resolution mass spectrometry, LC: Liquid chromatography, LC-EC: liquid chromatography–electrochemical detection, LLE: Liquid–liquid extraction, LSE: Liquid–solid extraction, MAE: Microwave-assisted extraction, MeOH: Methanol, MP: Mobile phase, MS: Mass spectrometry, N/A: Not applicable, ND: Not detected, NMR: Nuclear magnetic resonance, o-PhA: orthophosphoric acid, PA: Perchloric acid, PDA: Photodiode array detector, PhA: Phosphoric acid, Ph. Buffer: Phosphate buffer, PHWE: pressurized hot water extraction, QTOF: Quadruple time-of-flight, SC-CO_2_: Supercritical carbon dioxide, SLE: solid–liquid extraction, SPD (DAD): Spectrophotometric detector, SPE: Solid-phase extraction, SWE: Subcritical water extraction, TFA: Trifluoroacetic acid, THF: Tetrahydrofuran, TQD: Tandem quadrupole detection, UAE: Ultrasound-assisted extraction, UHPLC: Ultra-High-Performance Liquid Chromatography, UV: Ultraviolet, Vis: Visible, VWD: Variable wavelength detector.

**Table 4:** Risk of bias assessment of the studies included in this review.

Domains					
Sample Selection & Matrix	Extraction Procedure	Analytical Technique/Instrumentation	Method Conditions & Parameters	Data, Calibration & Quantification	Reference
L	L	L	H	L	[81]
L	L	L	H	L	[82]
L	L	L	H	L	[83]
L	L	L	L	L	[84]
L	L	L	L	L	[85]
L	L	L	H	L	[86]
L	L	L	H	L	[87]
L	X	L	H	L	[88]
L	L	L	H	L	[89]
L	L	L	H	L	[90]
L	L	L	L	L	[91]
L	L	L	H	L	[92]
L	L	L	L	L	[93]
L	L	L	H	L	[94]
L	L	L	H	L	[95]
L	L	L	H	L	[96]
L	L	L	H	L	[97]
L	L	L	H	L	[98]
L	X	L	H	L	[99]
L	L	L	L	L	[100]
L	X	L	H	L	[101]
L	L	L	L	L	[102]
L	L	L	L	L	[103]
L	L	L	H	L	[104]
L	L	L	H	L	[72]
L	L	L	H	L	[47]
L	L	L	L	L	[105]
L	L	L	L	L	[106]
L	L	L	L	L	[28]
L	L	L	L	L	[107]
L	L	L	X	L	[108]
H	L	L	H	L	[73]
L	L	L	H	L	[109]
L	L	L	L	L	[110]
H	L	L	L	L	[111]
L	L	L	L	L	[25]
L	L	L	X	L	[112]
L	L	L	L	L	[113]
L	L	L	H	L	[114]
L	L	L	L	L	[115]
L	L	L	H	L	[116]
L	L	L	H	L	[117]
H	L	L	H	L	[71]
L	L	L	L	L	[118]
L	L	L	H	L	[119]
H	L	L	H	L	[70]
H	L	L	H	L	[120]
L	L	L	L	L	[121]
L	L	L	L	L	[122]
L	L	L	L	L	[123]
L	L	L	H	L	[124]
L	L	L	L	L	[125]
L	L	L	H	L	[126]
L	L	L	L	L	[78]
L	L	L	L	L	[127]
L	L	L	H	L	[37]
L	L	L	H	L	[128]
L	L	L	H	L	[129]
L	L	L	H	L	[130]
L	L	L	H	L	[131]
L	L	L	H	L	[64]
H	L	L	H	L	[20]
L	L	L	H	L	[21]
L	L	L	H	L	[79]
L	L	L	L	L	[53]
L	L	L	H	L	[34]
L	L	L	X	L	[5]
L	L	L	L	L	[16]
L	L	L	X	L	[30]
L	L	L	H	L	[45]
L	L	L	L	L	[56]
L	L	L	L	L	[32]
L	L	L	L	L	[39]
L	L	L	L	L	[48]
H	L	L	L	L	[74]
L	L	L	H	L	[24]
L	L	L	X	L	[12]
L	L	L	L	L	[13]
L	L	L	X	L	[46]
L	L	L	L	L	[132]
L	L	L	H	L	[36]
L	L	L	H	L	[42]
L	L	L	H	L	[57]
L	L	L	L	L	[76]
L	L	L	L	L	[65]
L	L	L	L	L	[58]
L	L	L	L	L	[63]
L	L	L	H	L	[75]
L	L	L	X	L	[60]
L	L	L	L	L	[23]
L	L	L	L	L	[59]
L	L	L	L	L	[35]
L	L	L	H	L	[44]
H	L	L	H	L	[77]
L	L	L	H	L	[54]
L	L	L	H	L	[27]
L	L	L	H	L	[22]
H	L	L	H	L	[133]
L	L	L	H	L	[62]
L	L	L	L	L	[19]
L	L	L	L	L	[15]
L	L	L	H	L	[134]
L	L	L	H	L	[135]
H	L	L	L	L	[136]
L	L	L	H	L	[38]
L	L	L	H	L	[33]
H	L	L	H	L	[137]
L	L	L	L	L	[138]
L	L	L	L	L	[31]
L	L	L	L	L	[17]
L	L	L	H	L	[11]
L	L	L	H	L	[43]
L	L	L	H	L	[29]
L	L	L	H	L	[139]
L	L	L	X	L	[80]
L	L	L	X	L	[26]
L	L	L	L	L	[41]
L	L	L	H	L	[14]
L	L	L	H	L	[140]
L	L	L	H	L	[141]
L	L	L	H	L	[142]
L	L	L	L	L	[143]
L	L	L	H	L	[144]
L	L	L	H	L	[145]

L = Low risk; H = High risk; X = Unclear risk.

## Data Availability

No new data were created or analyzed in this study. Data sharing is not applicable.

## Abbreviations

The following abbreviations are used in this manuscript:

3-CQA	3-Caffeoylquinic Acid
4-CQA	4-Caffeoylquinic Acid
5-CQA	5-Caffeoylquinic Acid
ATR	Attenuated Total Reflectance
CAD	Charged Aerosol Detector
CE	Capillary Electrophoresis
CGAs	Chlorogenic Acids
CZE	Capillary Zone Electrophoresis
DAD	Diode Array Detector
DES	Deep Eutectic Solvent
EC	Electrochemical Detection
ESI	Electrospray Ionization
FT-ICR	Fourier Transform–Ion Cyclotron Resonance
FT-MIR	Fourier Transform–Mid-Infrared
FQAs	Feruloylquinic Acids
GC-MS	Gas Chromatography–Mass Spectrometry
HPLC	High-Performance Liquid Chromatography
HPTLC	High-Performance Thin-Layer Chromatography
HRMS	High Resolution Mass Spectrometry
IR	Infrared Spectroscopy
LC-MS	Liquid Chromatography–Mass Spectrometry
LLE	Liquid–Liquid Extraction
LSE	Liquid–Solid Extraction
MAE	Microwave-assisted Extraction
MS/MS	Tandem Mass Spectrometry
NADES	Natural Deep Eutectic Solvents
NMR	Nuclear Magnetic Resonance
PDA	Photodiode Array Detector
PHWE	Pressurized Hot Water Extraction
PRISMA	Preferred Reporting Items for Systematic Reviews and Meta-Analysis
QTOF	Quadrupole Time-of-Flight
RT	Room Temperature
SCF	Supercooling-Facilitating
SC-CO_2_	Supercritical Carbon Dioxide Extraction
SLE	Solid–Liquid Extraction
SPE	Solid-Phase Extraction
SFE	Supercritical Fluid Extraction
SWE	Subcritical Water Extraction
TQD	Tandem Quadrupole Detection
UAE	Ultrasound-assisted Extraction
UPLC	Ultra Performance Liquid Chromatography
UV/Vis	Ultraviolet/Visible Absorption Spectroscopy
VAE	Vortex-Assisted Extraction
VWD	Variable wavelength detector
